# Leveling the Playing Field: A New Initiative to Publish Negative and Replication Data in Brain Trauma

**DOI:** 10.1089/neur.2020.0055

**Published:** 2020-10-29

**Authors:** Chantelle Ferland-Beckham, Sandra Petty, Eric Prager, Nicole Harmon, Magali Haas, Andreas Jeromin

**Affiliations:** ^1^ Cohen Veterans Bioscience, Cambridge, Massachusetts, USA.; ^2^ Center for Biomedical Research Transparency, New York, New York, USA.

Our scientific culture has an embedded ethos that publishing positive results equates to more success, productivity, interest, and *value*. This has become apparent as the proportion of positive results published in scientific literature has increased by 6% per year since 1990^1^ and, in neuroscience, estimates indicate that approximately 85% of manuscripts published include only positive results.^2^ Unfortunately, publishing *only* positive results has considerable consequences that significantly affect research progress. Indeed, when negative results (defined as studies that do not reach statistical significance or do not confirm expected results or working hypotheses) are withheld from the public record, scientific knowledge is skewed toward the positive results, many of which are irreproducible.^3^ This can lead to harmful interpretations of risk-benefit when clinical trial results remain unpublished^4^ or can negatively affect meta-analyses, leading to potentially biased conclusions for scientists, researchers, and policymakers.^5^ Failure to publish negative results delays scientific progress when researchers toil away in vain at ideas that are incorrect or flawed; this results in wasted time (especially when others repeat these failed experiments), effort, and money. At its most extreme, priority to publish positive results might lead researchers to resort to unethical approaches, including tweaking hypotheses that better suit the data,^6^ massaging data to draw conclusions that will appeal to prestigious research journals, or falsifying data.

Publishing well-designed negative studies and replication studies are valuable to the scientific ecosystem for a number of reasons: 1) publishing such results that may contradict established consensus can open dialogue for a new understanding of a particular question; 2) researchers have a more complete picture of the state of their specific field and can design their research plans accordingly; and 3) funders can divert funding from erroneous or flawed hypotheses toward potentially more successful endeavors. Most importantly, publishing well-designed negative studies and replicating previously published studies leads to transparent and well-balanced reporting, tenets that are central to rigorous and efficient experimental design, scientific advancement, and improving patient outcomes.

A scientific culture in which negative results or replicate studies are not valued exacerbates wastage and irreproducibility. Alternatively, knowing what didn't work as expected and adjusting the research plan improves transparency and reproducibility of research; this is good for science. It is therefore time to overcome the stigma of submitting well-designed negative results and replicate studies into the public domain, and to embrace a culture that accepts and supports publication of types of studies.

Igniting a cultural shift in the scientific ecosystem begins with publishing well-designed studies where outcomes do not confirm expected results or the working hypothesis. To this end, *Neurotrauma Reports Null Hypothesis* is a special collection of articles that will be published in *Neurotrauma Reports*. It will be comprised of high-quality, well-conducted, peer-reviewed studies that incorporate negative, inconclusive, or replication findings in the field of brain trauma. A joint initiative sponsored by the Center for Biomedical Research Transparency (CBMRT) and Cohen Veterans Bioscience (CVB)—organizations with missions to promote transparent reporting—*Neurotrauma Reports Null Hypothesis* will launch during the 10^th^ Annual Traumatic Brain Injury Conference.

A recently announced call for papers, to be published as an ongoing series, is open to well-performed negative, inconclusive, and replicative studies within the scope of *Neurotrauma Reports*, including but not limited to, traumatic brain injury, stroke, and spinal cord injury. Studies should be full-length empirical studies, adhere to applicable research rigor standards, include an appropriate description of the methodology, and clearly describe statistical analyses and approaches used to support conclusions. Researchers who submit studies for publication in this special collection may also include extended supplementary materials.

Changing the scientific ecosystem to ensure all well-conducted studies are considered, no matter the outcome, is a much-needed culture shift that will reduce bias, improve research practices, and will lead to a greater impact. It is our hope that with this collection we will engage the broader scientific community to ensure best practices are followed, and that by including all findings, scientific research will progress faster leading to a better understanding of the biological underpinnings of brain injury related disorders and the development of personalized therapeutic approaches.

## Abbreviations Used

CBMRT: Center for Biomedical Research Transparency
CVB: Cohen Veterans Bioscience
